# Identification of a rare *COCH* mutation by whole-exome sequencing: Implications for personalized therapeutic rehabilitation in an Austrian family with non-syndromic autosomal dominant late-onset hearing loss

**DOI:** 10.1007/s00508-017-1230-y

**Published:** 2017-07-21

**Authors:** Thomas Parzefall, Alexandra Frohne, Martin Koenighofer, Andreas Kirchnawy, Berthold Streubel, Christian Schoefer, Wolfgang Gstoettner, Klemens Frei, Trevor Lucas

**Affiliations:** 1 Department of Otorhinolaryngology, Head and Neck Surgery, Medical University of Vienna, Waehringer Guertel 18–20, 1090 Vienna, Austria; 2 Department for Cell and Developmental Biology, Orphan disease genetics group, Center for Anatomy and Cell Biology, Medical University of Vienna, Vienna, Austria; 3 Clinical Institute of Pathology, Medical University of Vienna, Vienna, Austria

**Keywords:** Non-syndromic hereditary hearing loss, Autosomal-dominant hearing loss, Whole-exome sequencing, Cochlin, Cochlear implant

## Abstract

**Background:**

Non-syndromic autosomal dominant hearing impairment is characteristically postlingual in onset. Genetic diagnostics are essential for genetic counselling, disease prognosis and understanding of the molecular mechanisms of disease. To date, 36 causative genes have been identified, many in only individual families. Gene selection for genetic screening by traditional methods and genetic diagnosis in autosomal dominant patients has therefore been fraught with difficulty. Whole-exome sequencing provides a powerful tool to analyze all protein-coding genomic regions in parallel, thus allowing the comprehensive screening of all known genes and associated alterations.

**Methods:**

In this study, a previously undiagnosed late-onset progressive autosomal dominant hearing loss in an Austrian family was investigated by means of whole-exome sequencing. Results were confirmed by Sanger sequencing.

**Results:**

A previously described c.151C>T missense (p.Pro51Ser) mutation in the LCCL (limulus factor C, cochlin, late gestation lung protein Lgl1) domain of the cochlin gene (*COCH*) was identified as causative and segregated with disease in five members of the family. Molecular diagnostics led to the decision to perform cochlear implantation in an index patient who subsequently showed excellent postoperative auditory performance. The c.151C>T mutation was not found in 18 screened Austrian families with autosomal dominant hearing loss but was represented alongside other known pathogenic mutant *COCH* alleles in the Genome Aggregation Database (gnomAD) in European populations. A combined allele frequency of 0.000128 implies an orphan disease frequency for *COCH*-induced hearing loss of 1:3900 in Europe.

**Conclusions:**

Exome sequencing successfully resolved the genetic diagnosis in a family suffering from autosomal dominant hearing impairment and allowed prediction of purported auditory outcome after cochlear implantation in an index patient. Personalized treatment approaches based on the molecular mechanisms of disease may become increasingly important in the future.

## Introduction

Hereditary hearing impairment (HI) is a heterogenous disease that can vary in degree of disability, time of onset, accompanying symptoms, mode of inheritance and underlying molecular pathomechanism. The most common form is newborn non-syndromic HI that is mostly recessively inherited and in Caucasians is caused by alterations in the gap junction beta 2 (*GJB2*) gene in approximately 50% of cases [1]. Dominant forms of genetic HI amount to almost 20% of cases. In the latter group, HI usually develops at different stages of life and gradually progresses over time. Unlike congenital recessive forms, no common causative genes have been identified for dominant late-onset progressive HI, making gene selection for genetic screening of dominant cases particularly challenging.

In recent years, the application of massively parallel sequencing (MPS) has greatly accelerated and improved the diagnosis of Mendelian diseases [2]. To reduce the costs of analysis and sequencing data loads, targeted capture of known HI genes and whole-exome sequencing (WES) have been successfully applied to identify causative genes and alterations in HI cohorts from numerous populations [3–5].

Hearing rehabilitation in affected patients is achieved by hearing aid adaptation or, in severe and profound cases, by means of cochlear implantation (CI). This procedure is highly standardized and successful in congenital HI cases in young children. In recent years, the indication spectrum for CI has expanded to postlingual deaf adult individuals, although auditory performance with the bionic ear differs more widely in this patient group and is less predictable [6]. Several studies have addressed CI performance predictability in both pediatric and adult CI recipients and some genetic factors were found to be associated with either good [7–9] or poor [10] auditory performance, leading to speculation that particular patients with altered gene expression in retrocochlear neuronal structures could be less suitable candidates for implantation [11]. A common finding in many patients with HI is that high frequencies are affected earlier in time and more severely, while low and mid frequencies are still preserved or less affected. Electroacoustic stimulation (EAS) cochlear implants take advantage of a combinatory approach for hearing rehabilitation using a short cochlear implant electrode to stimulate only the basal turns that transduce high frequency signals while leaving apical cochlear regions intact to allow natural acoustic stimulation [12].

In this study, we applied WES to investigate the genetic cause of disease in four generations of an Austrian family suffering from dominant very-late-onset progressive HI. We also demonstrate by a case report of one of the family members how the molecular diagnostics helped to predict cochlear implant performance and aided in the clinical decision to perform bilateral EAS cochlear implant surgery in the patient.

## Patients and methods

### Patients and clinical test battery

The patients participating in this study were recruited at the department of Otorhinolaryngology at the Medical University of Vienna, Austria. The study met the World Medical Association (WMA) Helsinki Declaration criteria and was approved by the local Ethics Committee of the Medical University of Vienna (ECS 198/2004, last extension: January 2017). After obtaining informed consent, clinical and audiometric examinations were performed on all participants. The test battery included ear inspection, interrogation of full medical history (family medical history, age of onset, accompanying symptoms, history of noise trauma or exposure to ototoxic drugs) and pure tone audiometry. The Freiburger monosyllabic speech test was used to determine word recognition scores. The Freiburger speech audiometry test consists of two independent test series that present a list of two digit numbers and a list of monosyllabic words to the patient. To measure speech intelligibility a) the intensity level needed to understand 50% of two-digit numbers correctly (speech recognition threshold; SRT), b) the percentage of monosyllabic words understood correctly at 65 dB (65 dB phenome score) and c) at 95 dB intensities (95 dB phenome score) are obtained. In normal hearing individuals, the SRT is 18.4 dB and a 100% score of correctly identified monosyllables is achieved at 50 dB. Additionally, in the patient receiving a CI, high-resolution temporal bone computed tomography (CT) and magnetic resonance imaging (MRI) of the brain and cochlea was performed prior to surgery. The index patient family suffering from late-onset progressive HI was termed family AD1.

### DNA sequencing

Patient whole blood was drawn from a peripheral vein and genomic DNA was then extracted with a commercial DNA isolation kit (Invisorb blood universal kit 1000, STRATEC Molecular, Berlin, Germany). Initially, all patient samples were prescreened for variants in the *GJB2* coding region as described previously [13]. An index patient from family AD1 (member III/7) was then selected for WES.

Whole-exome libraries were created with a commercial capture kit (SureSelectXT All Exon, V5, Agilent Technologies, Santa Clara, CA) according to the manufacturer’s instructions and enriched samples then underwent paired-end sequencing on a HiSeq 2000 device (Illumina Biotechnology, San Diego, CA). A cochlin variant identified in the index patient by WES was then validated in the remaining family members for co-segregation and in single index patients from a cohort of 18 Austrian families suffering from dominant bilateral progressive HI with Sanger-based sequencing. For validation PCR, forward 5’-CAGAGGCTTGGACATCAGGA-3’ and reverse 5’-ACGTCTGCATTTCTCTCCCA-3’ primers were used.

### Bioinformatics

Read mapping to the human reference genome (version hg19) and variant calling were performed using the Burrows-Wheeler read aligner [14] and the Genome Analysis Tool Kit [15], respectively. All single nucleotide variants, deletion and insertion variants in coding regions and splice sites along with 5 bp upstream and downstream of the adjacent intronic sequences were uploaded to an online MPS analysis platform (Genomatix GeneGrid, Genomatix, Munich, Germany) for further investigation. Filters were applied to exclude any variants with an allele frequency higher than 0.01 in the gnomAD database [16] and only heterozygous variants matching the dominant inheritance in family AD1, were included.

### Cochlear implantation

Bilateral cochlear implantation was performed with a MedEl Synchrony® device (MedEl Medical Electronics, Innsbruck, Austria) coupled to a 20 mm flex EAS electrode inserted into the cochlea via a round window approach on the left and a promontorial cochleostomy on the right side.

## Results

**Fig. 1:**
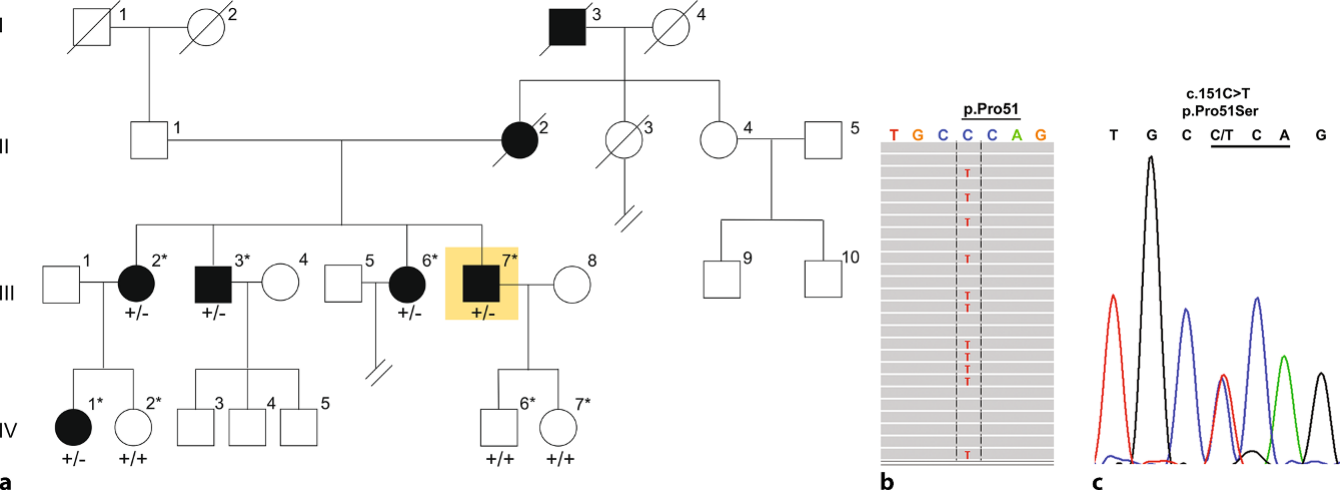
**a** Autosomal dominant pedigree of the hearing-impaired (*black*) family under study showing the individual examined by whole-exome sequencing (III/7, *shaded yellow*). All affected (III/2, III/3, III/6, IV/1) and unaffected (IV/2, IV/6, IV/7) siblings available to the study are marked with an asterisk (*). **b** Representative graphic of the 35×C and 36×T whole-exome sequencing reads at *COCH* c.151 in affected proband III/7 visualized with Integrative Genomics Viewer software and **c** representative chromatogram showing heterozygous (+/−) inheritance of c.151C>T (p.Pro51Ser) in an affected family member by Sanger sequencing (codon 51 is *underlined*)

The index family AD1 is a 4-generation Austrian family suffering from progressive sensorineural non-syndromic HI. The eight family members signed informed consent and were available to the study including five affected (III/2, aged 68 years; III/3, aged 67; III/6, aged 65; III/7, aged 61; IV/1, aged 45) and three non-affected (IV/2, aged 41 years; IV/6, aged 28; IV/7, aged 24) family members (Fig. 1).

### Mapping and whole-exome sequencing results

Paired-end sequencing of the capture library of the index proband (III/7) rendered an average total read number of 8.27 Mbp. The median base coverage was 49x, with 92.2% and 73.9% of targeted bases covered by more than 10 or 30 reads, respectively. Variant analysis in the index patient (III/7) revealed a heterozygous missense c.151C>T mutation in the cochlin gene (*COCH*) that has been previously reported to cause progressive late-onset HI in Dutch, Belgian and US families ([17–21]; Fig. 1). This single nucleotide alteration leads to an amino acid exchange from proline to serine at position 51 of the mature cochlin peptide (p.Pro51Ser). Sanger sequencing demonstrated that the c.151C>T mutation co-segregated with the HI phenotype in all family AD1 members and was fully penetrant (Fig. 1). Genetic screening of a cohort of index patients from 18 further families with dominant sensorineural HI to test the frequency of this variant in our patient collective did not identify the c.151C>T variant in any of the patients.

### Clinical observations in family AD1

**Fig. 2:**
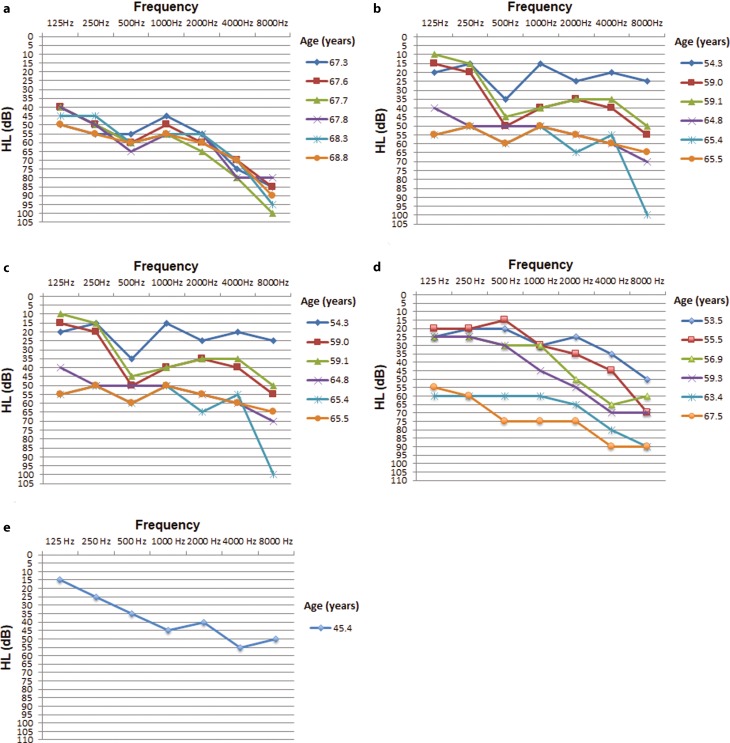
Unaided, masked pure tone audiograms in dB at different ages (years) of affected family members III/2 (**a**), III/3 (**b**) III/6 (**c**), III/7 (**d**) and IV/1 (**e**) show very-late-onset progressive hearing loss (single ear shown)

The onset of HI occurs in the 4th to 5th decade of life with an initial drop at high frequencies that gradually expands to pantonal profound HI by the end of the 6th decade (Fig. 2). Freiburger speech test results were available from 4 patients and showed severe speech recognition threshold (SRT) shifts and speech discrimination loss in the patients over 60 years of age (Table 1). Additionally, the index patient (III/7) presented with bilateral vestibular areflexia (data not shown).

**Table 1:** Speech audiometry in family AD1 determined by unaided Freiburger monosyllabic word testing, with bilateral hearing aids and 6 months after cochlear implant surgery

ID^a^	Gender	Age (years)	Ear (side)	SRT(dB)	Phenome score at 65 dB (%)	Phenome score at 95 dB (%)
IV/1	F	45	R	7	100	100
			L	29	95	95
III/2	F	67.3	R	63	0	30
			L	87	0	0
		68.3	R	57	0	30
			L	>110	0	0
III/6	F	65.4	R	51	0	70
			L	39	30	45
III/7	M	55.6	R	70	0	80
			L	65	25	80
		59.0	R	68	0	30
			L	60	0	40
		60.6	R	95	0	15
			L	85	0	35
		61.2	R	71	0	20
			L	58	0	35
*With hearing aids before cochlear implant (III/7)*						
–	–	61.2	R	n. t.	20	n. t.
			L	n. t.	20	n. t.
*6 months after bilateral electroacoustic cochlear implant (III/7)*						
–	–	62.8	R	35	60	n. t.
			L	40	60	n. t.

*F* female, *M* male, *dB* decibel, *SRT* speech recognition threshold = 50% double digit numbers correct, *n. t.* not tested, *R* right ear, *L* left ear

^a^Speech audiometry results of patient III/3 were not available

### Auditory performance before and after bilateral EAS cochlear implantation in the index patient III/7

**Fig. 3:**
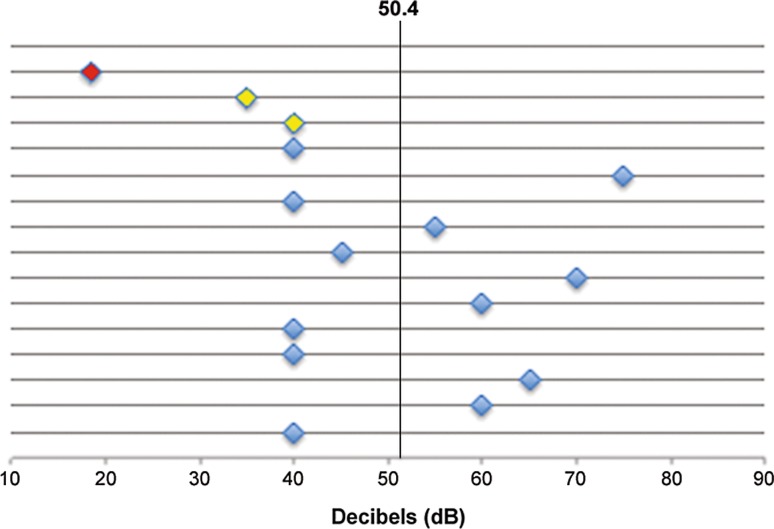
Speech recognition thresholds in a cohort of 15 age-matched patient ears receiving electroacoustic cochlear implants between 2008 and 2016 at the department of Otorhinolaryngology, Head and Neck Surgery, Medical University of Vienna. The index patient in this study (III/7) is shown in *yellow* (left and right ear). For reference, the speech recognition threshold in an average normal hearing adult is displayed in *red*. The *vertical line* displays the mean SRT of 50.4 dB in the cohort under investigation

Pure tone audiometric tests 6 months after EAS cochlear implantation in the index patient III/7 resulted in a pure tone average of 35 dB HL in the right and 40 dB in the left ear compared to 71 and 65 dB HL, respectively, before cochlear implantation (data not shown). Speech intelligibility after EAS CI as measured by speech audiometry was exceptionally good, reaching 50% correct double digit numbers (=SRT) at 35 dB and 40 dB in the right and left ears, respectively. For reference, in an age-matched sample of 15 ears implanted with EAS cochlear implants between 2008 and 2016 the corresponding mean sound intensity at which 50% of double digit numbers were reproduced correctly was 50.4 dB (range 35–75 dB) (Fig. 3). At 65 dB the patient III/7 reached a phenome score of 60% correct monosyllables bilaterally compared to 20% correct monosyllables bilaterally with the best possible hearing aid amplification before surgery (Table 1).

## Discussion

In the present study, a family with progressive non-syndromic autosomal dominant HI of unknown genetic etiology was analyzed for the causative mutation by means of WES and a known pathogenic c.151C>T missense transversion within the *COCH* gene was identified. Molecular diagnostics in patients with autosomal dominant forms of HI are particularly challenging because no major, frequent causative genes have been identified to date in this patient group; however, WES allowed an effective and rapid diagnosis in family AD1. The strength of WES as a comprehensive screening and effective diagnostic tool in dominant HI patients is therefore emphasized by our findings.

This is the first family reported in Austria with a *COCH* mutation causing non-syndromic dominant HI. Our findings imply that *COCH* should be included in the genetic screening for autosomal dominant HI in Austria, though extending the screening for c.151C>T to a cohort of index patients from 18 families with autosomal dominant sensorineural HI did not identify this mutation in any of the patients under study.

Cochlin is a secretory protein that is encoded by the *COCH* gene located at 14q11.2-q13. The full length protein is transcribed from 12 exons and comprises 550 amino acids that form a signal peptide, an LCCL (limulus factor C, cochlin, late gestation lung protein Lgl1) domain, which is thought to bind lipopolysaccharides and to possibly play a role in innate host defence mechanisms [22, 23] and two von Willebrand factor type A (vWFA) homology domains, which are ligand-binding domains often involved in collagen interactions and predominantly found in secreted proteins [24]. Numerous isoforms of cochlin are expressed, which emerge from alternative splicing and posttranslational processing. The full length protein is uniquely and highly expressed in the inner ear [25–27], particularly in the habenula perforata, in fibrocytes of the spiral limbus, spiral ligament, modiolus and beneath the sensory epithelium of the cristae ampullaris, in the maculae and in vestibular nerve fiber channels [28–30].

The *COCH*-associated hearing phenotype (autosomal-dominant deafness type 9; DFNA9) is linked to cochlear abnormalities that are characterized by the accumulation of large amounts of cochlin-containing, acidophilic deposits in the area of the spiral limbus, spiral ligament, spiral osseous lamina, the stroma of cristae and maculae and vestibular nerve channels [28]. Cochlin null mice exhibit normal hearing, which is suggestive of a dominant-negative or gain-of-function disease mechanism [31]. It is conceivable that the deposits seen in DFNA9 disturb inner ear function and integrity leading to gradual cell degeneration and progressive hearing deterioration.

The c.151C>T (NM_001135058) mutation found in the presented family leads to the replacement of a highly conserved proline with a serine. Nuclear magnetic resonance spectroscopy (NMR) has revealed that Pro51 is located on the surface of the wild-type protein. The c.151C>T mutation plays a direct role in protein folding in vitro and prevents the generation of 3D structures characteristic of the wild-type LCCL domain [32]. These results suggest that pathological effects from the p.Pro51Ser mutation may stem from a misfolded protein phenotype rather than from protein instability or altered interactions with hypothetical binding partners. Transfection of human cell cultures with transiently expressed p.Pro51Ser mutant proteins did not result in intracellular accumulation and extracellular distribution patterns appeared normal compared to wild-type proteins; however, the proteins might behave differently within the native ECM of the inner ear or over longer periods of time [33, 34]. It has been shown that mouse mutant proteins corresponding to human p.Pro51Ser form stable, secreted homo-dimers and even homo-oligomers with wild-type cochlin.; however, early dimers are not detectable under reducing conditions that dissolve disulfide bonds. Thus, abnormal disulfide pairing has been proposed to lead to dimerization. Injection of these dimeric cochlin complexes into the mouse inner ear has confirmed damaging effects [35]. Not only could these interactions account for a dominant-negative effect, it is also conceivable that the misfolded oligomers accumulate to form the deposits characteristic of DFNA9.

The p.Pro51Ser mutation had already been detected in Dutch, Belgian and American families and is believed to result from a founder effect. The reported phenotypes are consistent as to the age of onset (mostly in the fifth decade of life), progressive nature starting at high frequencies and the occurrence of vestibular manifestations with incomplete penetrance [17–21]. Initial high frequency hearing loss observed in the present patient and previously described DFNA9 patients and the very late onset in the fifth decade of life resemble the findings in patients with age-related hearing loss. It is therefore possible that genetic variability in the Cochlin gene may play a role in this condition as well. To date, 21 pathogenic mutations in *COCH* have been reported [36]. Analysis of non-Finnish European allele frequencies in the gnomAD database [16] revealed the presence of p.Pro51Ser (frequency; 0.000009), p.Pro89His (0.00011) and p.Met512Thr (0.000009). A combined mutated allele frequency of 0.000128 indicates that 2.56 subjects in a population of 10,000 bear a heterozygous pathogenic *COCH* mutation in Europe.

The full length cochlin isoform is exclusively expressed in the inner ear and patients with mutations in *COCH* do not show further phenotypes despite inner ear pathologies. Furthermore, patients with *COCH* mutations have been previously reported to have good auditory outcomes after cochlear implantation [9]. The molecular diagnosis in family AD1 allowed an estimation of expected auditory performance after cochlear implantation in the index patient III/7. This was important in the clinical decision making in the patient because adult onset patients differ widely in outcomes after CI. In fact, patient III/7 showed excellent bilateral speech recognition thresholds and speech discrimination values 6 months postoperatively. In addition, residual hearing could be well preserved in the patient. Although our findings only report the outcome data of a single patient, this case report illustrates how an improved personalized molecular diagnostic could routinely influence therapeutic decisions in personalized medicine in the near future.

## Conclusion

The use of WES successfully and rapidly identified the cause of disease in an Austrian family suffering from autosomal dominant progressive HI. This is the first report of a *COCH* mutation as the cause for HI in Austria and our findings extend the spectrum for genetic screening in this patient group in Austria. The molecular diagnostics allowed estimation of predicted outcome after cochlear implantation in an index patient. Further improvement in sequencing chemistry and genetic analysis pipelines in the future are likely to accelerate the diagnostic speed and advance of personalized treatment strategies.
